# ROS–NLRP3 Inflammasome Axis in Obesity: A Critical Review of Oxidative Stress-Driven Inflammation and Emerging Therapeutic Strategies

**DOI:** 10.3390/medicina62101969

**Published:** 2026-10-12

**Authors:** Sungeun Ahn, SangMin Park, Shakina Yesmin Simu

**Affiliations:** 1 Department of Biopharmaceuticals, Catholic Sangji University, Andong 36686, Republic of Korea; seahn@csj.ac.kr (S.A.); smpark@csj.ac.kr (S.P.); 2 College of Pharmacy, Gachon University, Incheon 21936, Republic of Korea; 3 Department of Oriental Medicinal Biotechnology, College of Life Sciences, Kyung Hee University, Yongin 17104, Republic of Korea

**Keywords:** oxidative stress, reactive oxygen species, mitochondrial ROS, NLRP3 inflammasome, obesity, insulin resistance, chronic inflammation, TXNIP

## Abstract

*Background and Objectives:* Obesity is a chronic, low-grade inflammatory state. Oxidative stress and reactive oxygen species (ROS) are increasingly recognized as not just passive byproducts but also signaling contributors to metabolic dysfunction in obesity. Among the inflammatory effectors that interact with ROS, the NOD-, LRR-, and pyrin domain-containing protein 3 (NLRP3) inflammasome has emerged as an important node linking metabolic stress to interleukin (IL)-1β/IL-18-mediated inflammation and insulin resistance. This review critically examines the ROS–NLRP3 axis as a mechanistic framework for obesity progression, weighing supporting against contradictory evidence and defining translational opportunities. *Materials and Methods:* A narrative concept-driven review was conducted by searching PubMed, Scopus, and Web of Science throughout August 2026, prioritizing mechanistic, human, and interventional studies of ROS, mitochondrial ROS (mtROS), NLRP3 activation, and obesity, with deliberate inclusion of discordant findings. *Results:* ROS-dependent NF-κB signaling contributes to NLRP3 priming, whereas mtROS, oxidized mitochondrial DNA, and thioredoxin-interacting protein (TXNIP) are consistently implicated in NLRP3 activation and caspase-1-dependent maturation of IL-1β and IL-18, which are associated with adipose tissue inflammation and impaired insulin signaling. However, loss-of-function studies have yielded inconsistent metabolic protection; human evidence is largely observational, and NLRP3 inhibitors, such as MCC950, carry organ-specific toxicities, indicating context dependence rather than a simple linear pathway. Upstream (mitochondria-targeted antioxidants, TXNIP blockade), core (selective NLRP3 inhibitors), and downstream (IL-1β/IL-18 blockade) strategies are supported mainly by preclinical or early clinical data; for metformin, SGLT2 inhibitors, and GLP-1 receptor agonists, ROS/NLRP3 modulation is a potentially relevant contributory mechanism. *Conclusions:* The ROS–NLRP3 axis provides a useful mechanistic framework for obesity-associated inflammation, but human causal evidence is limited, and its contribution is tissue-, stage-, and context-dependent; therefore, human-validated, mechanism-based studies are needed.

## 1. Introduction

Obesity is one of the most pressing global public health challenges, affecting more adults than undernutrition, and its incidence continues to increase across most regions [1,2]. It arises from a chronic imbalance between energy intake and expenditure that drives adipocyte hypertrophy and hyperplasia and is a major risk factor for type 2 diabetes mellitus (T2DM), cardiovascular disease, metabolic dysfunction-associated steatotic liver disease (MASLD; previously termed non-alcoholic fatty liver disease, NAFLD), and several cancers [3,4,5]. Rather than recataloging the well-established epidemiology and multifactorial etiology of obesity, this review treats it as an established context and directs its analytical focus to the mechanistic core that connects metabolic overload to disease: oxidative stress and its downstream inflammatory effectors.

A defining feature of obesity is chronic low-grade inflammation sustained by dysfunctional adipose tissue. Central to this state is the overproduction of reactive oxygen species (ROS), particularly mitochondrial ROS (mtROS), which accumulate when excess nutrients overwhelm mitochondrial and antioxidant capacity [6,7]. Historically, ROS were regarded largely as damaging byproducts; however, a growing body of work now positions them as signals that shape metabolic and inflammatory outcomes [8]. Among the inflammatory platforms that ROS engage, the NOD-, LRR-, and pyrin domain-containing protein 3 (NLRP3) inflammasome is among the most extensively studied in metabolic disease, converting oxidative and metabolic danger signals into mature interleukin (IL)-1β and IL-18 [9,10].

Existing reviews have largely examined these two areas of research in parallel: either focusing on oxidative stress in obesity or cataloging the NLRP3 inflammasome biology across metabolic tissues. Even recent syntheses connecting mitochondrial dysfunction to NLRP3 tend to present concordant findings of a linear cause-and-effect pathway [11]. However, three key limitations remain. First, ROS are usually framed as tissue damage rather than as a signal that contributes, through distinct mechanisms, to NLRP3 priming and activation. Second, a substantial body of contradictory evidence, most notably, the failure of several NLRP3-deficient rodent models to show metabolic protection, is rarely confronted, leaving the axis described as uniformly causal. Third, therapeutic discussion is typically compound-by-compound, rather than mapped onto the mechanistic node that engages each agent. The central thesis of this review is that ROS contribute to NLRP3 inflammasome regulation at two separable steps: ROS-dependent NF-κB signaling can support priming (signal 1), whereas mitochondrial dysfunction, mtROS, oxidized mtDNA, and TXNIP are implicated in activation (signal 2), and that the resulting ROS–NLRP3 axis, including a proposed feed-forward loop, provides a useful but context-dependent framework for obesity progression. Building on this thesis, the review makes four contributions that distinguish it from prior work: (i) it distinguishes ROS-dependent priming from mtROS-associated activation and integrates this two-step logic of NLRP3 with adipose-tissue pathophysiology (mtROS, oxidized mtDNA, TXNIP-dependent switching, and macrophage–adipocyte crosstalk); (ii) it critically weighs supporting against contradictory evidence, reconciling the discordance between preclinical loss-of-function models and human data rather than compiling only concordant findings; (iii) it argues that the axis is context-dependent rather than uniformly causal, and uses that nuance to explain both variable clinical results and the risks of non-selective suppression; and (iv) it converts this mechanistic reading into a three-level therapeutic map: upstream (ROS generation), core (inflammasome assembly), and downstream (cytokine effectors), with established metabolic drugs considered separately as agents for which ROS/NLRP3 modulation is a possible contributory mechanism, clarifying where mechanism-based intervention is most likely to translate.

## 2. Materials and Methods

**Figure 1:**
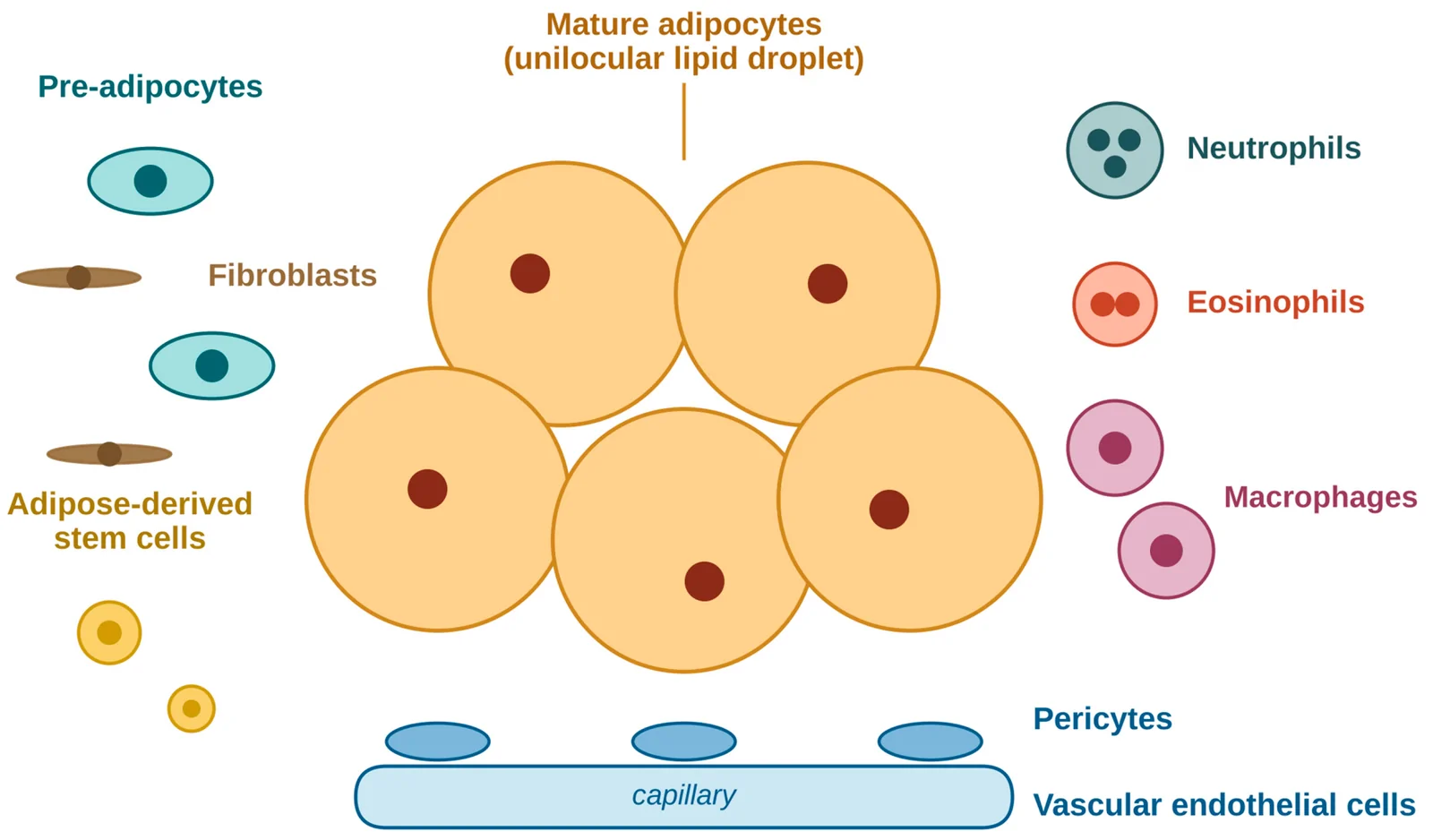
Cellular composition of adipose tissue. Adipose tissue comprises adipocytes and a stromal vascular fraction of preadipocytes, fibroblasts, vascular endothelial cells, pericytes, adipocyte-derived stem cells, and immune cells including macrophages, eosinophils, and neutrophils. Under chronic energy surplus, excess lipid storage drives adipocyte hypertrophy, immune-cell infiltration, and a pro-inflammatory, pro-oxidant microenvironment.

**Figure 2:**
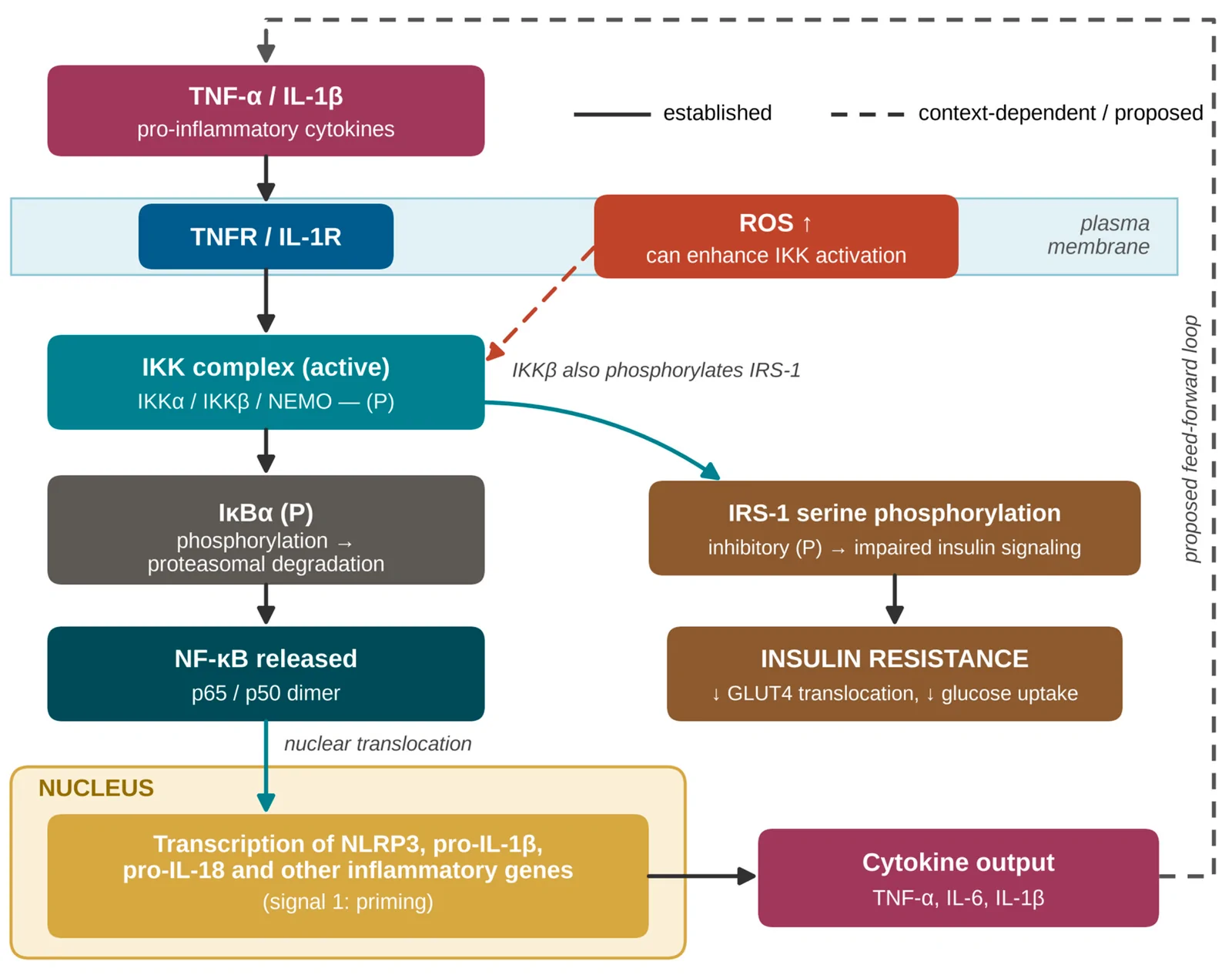
ROS-mediated canonical NF-κB signaling pathway in obesity. Under obesity-related oxidative stress, pro-inflammatory cytokines (TNFα, IL-1β) engage their receptors and activate the IKK complex, which phosphorylates IκBα and triggers its degradation, releasing NF-κB to drive the transcription of pro-inflammatory cytokines and of NLRP3, pro-IL-1β, and pro-IL-18 (priming). Sustained IKK–NF-κB activity promotes inhibitory serine phosphorylation of IRS-1. Solid arrows indicate established steps; dashed arrows indicate ROS-dependent enhancement and the proposed feed-forward loop, which are context-dependent.

**Figure 3:**
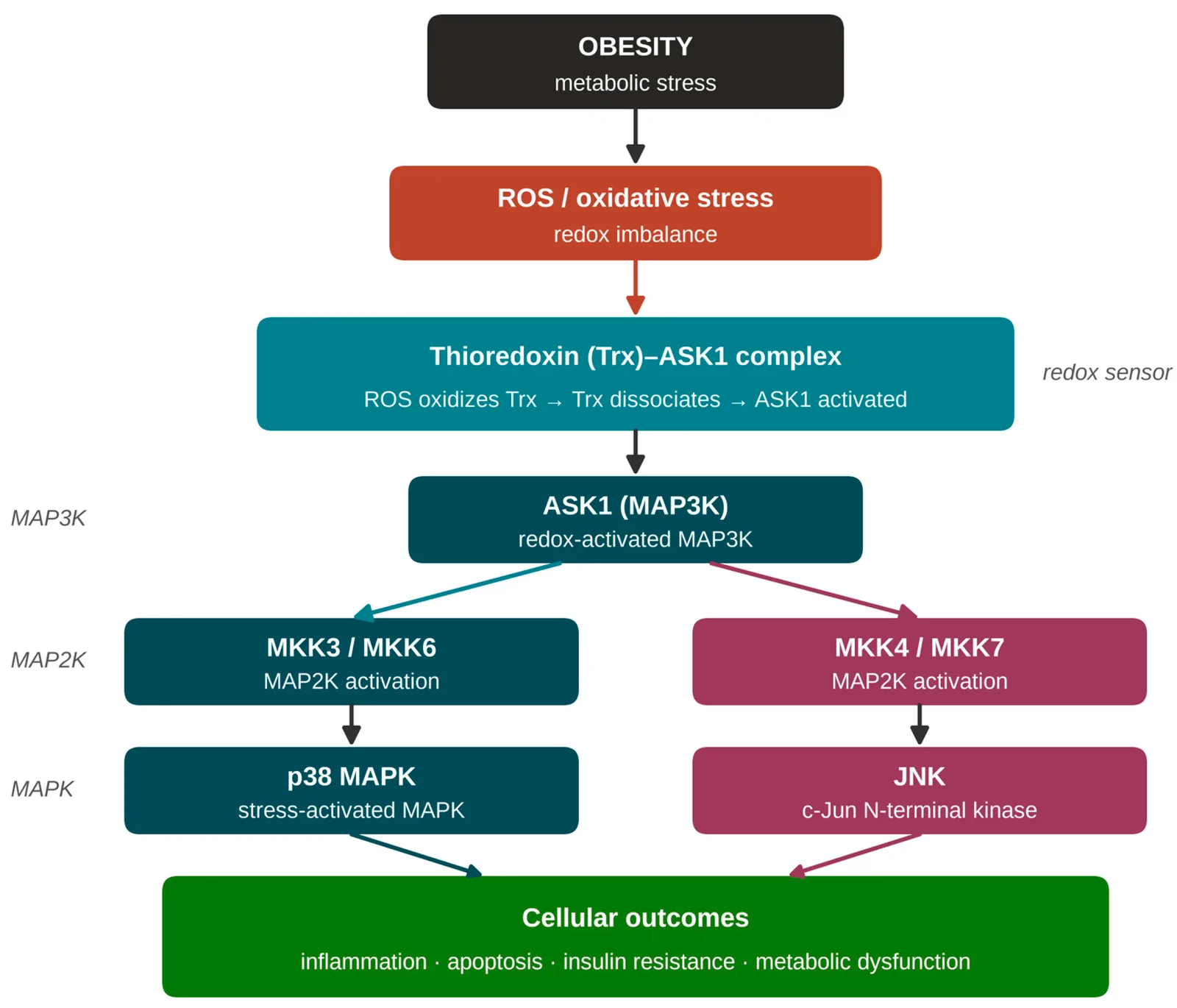
ROS-mediated activation of the MAPK signaling pathways during obesity. Increased intracellular ROS oxidizes thioredoxin, releasing and activating ASK1, which functions as an upstream MAP3K to phosphorylate MKK3/6 (p38 MAPK) and MKK4/7 (JNK). Sustained p38 MAPK and JNK signaling contributes to inflammation, apoptosis, and metabolic dysfunction.

**Figure 4:**
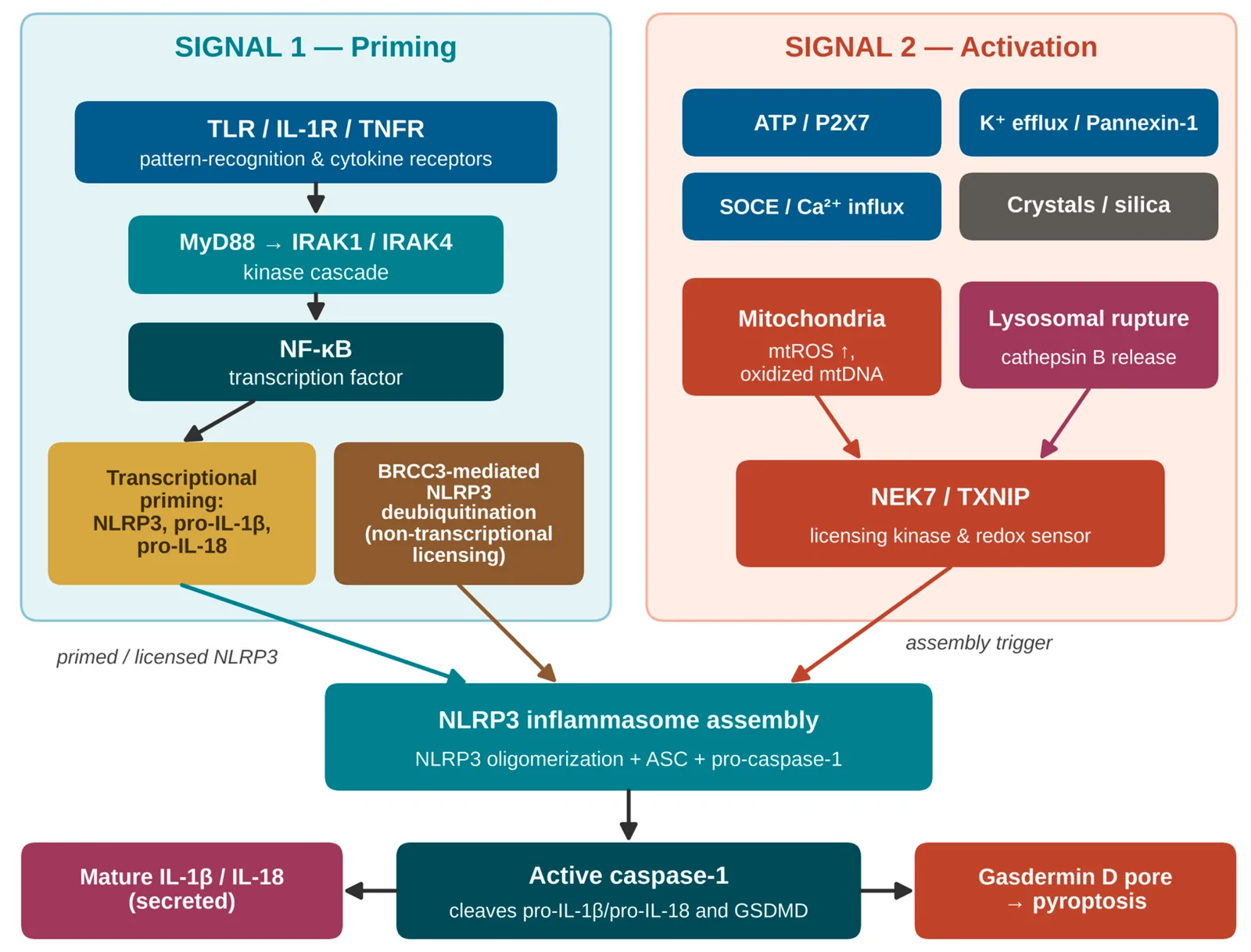
NLRP3 inflammasome activation follows a two-signal model. Signal 1 (priming) is initiated by microbial or endogenous stimuli through pattern-recognition and cytokine receptors, leading to MyD88-dependent NF-κB activation and transcription of NLRP3, pro-IL-1β, and pro-IL-18; BRCC3 (BRCC36)-mediated deubiquitination of NLRP3 provides non-transcriptional licensing. Signal 2 (activation) is triggered by potassium efflux, calcium flux, lysosomal destabilization, and mitochondrial signals (mtROS and oxidized mtDNA), together with TXNIP and NEK7, resulting in NLRP3 oligomerization, ASC recruitment, and caspase-1 activation.

**Figure 5:**
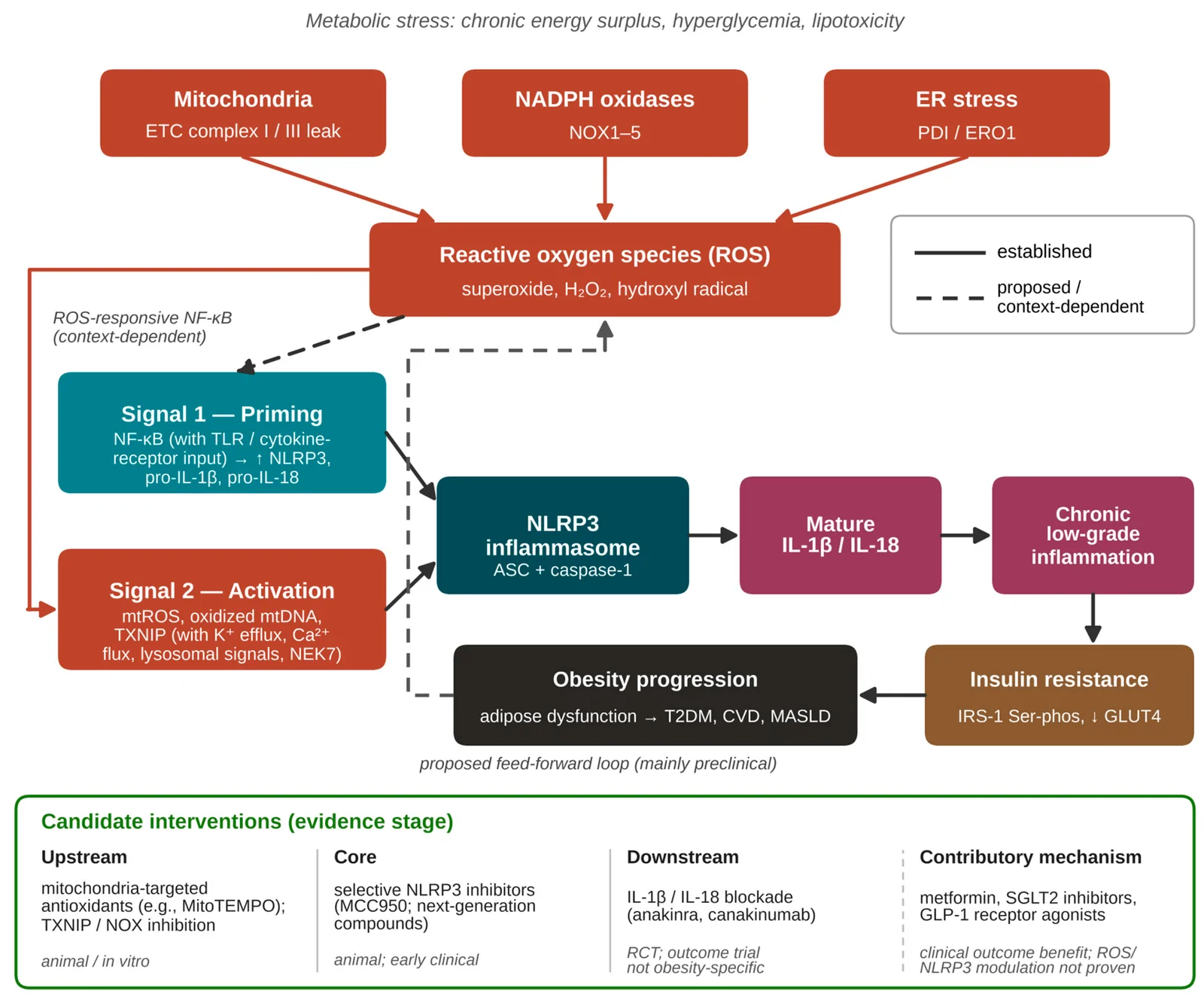
Proposed ROS–NLRP3 inflammasome axis in obesity progression (integrated summary). Nutrient overload and adipose expansion increase ROS from mitochondria, NADPH oxidases, and ER stress. Signal 1 (priming): ROS-responsive NF-κB signaling, together with TLR and cytokine-receptor inputs, increases NLRP3, pro-IL-1β, and pro-IL-18 expression. Signal 2 (activation): mtROS, oxidized mtDNA, and TXNIP, together with ionic and lysosomal signals, promote NLRP3 inflammasome assembly and caspase-1-dependent maturation of IL-1β and IL-18. The resulting chronic inflammation is associated with IRS-1 serine phosphorylation and insulin resistance, which may feed back to sustain metabolic stress and ROS. Candidate interventions are shown at upstream (mtROS scavengers, TXNIP), core (selective NLRP3 inhibitors), and downstream (IL-1β/IL-18 blockade) levels; metformin, SGLT2 inhibitors, and GLP-1 receptor agonists are shown separately as agents for which ROS/NLRP3 modulation is a possible contributory mechanism. Solid arrows indicate relationships established in experimental models; dashed arrows indicate proposed or context-dependent relationships, including the feed-forward loop.

This work is a narrative, concept-driven review rather than a systematic review. The literature was selected to construct and critically test a mechanistic argument rather than to catalogue all available studies. PubMed, Scopus, and Web of Science were searched for articles published until August 2026. For this revision, the PubMed search was rerun on 24 September 2026, with a publication-date limit of 31 August 2026, using six combinations with all terms restricted to title/abstract: (Q1) (obesity OR obese) AND (NLRP3 OR “NLRP3 inflammasome”) AND (“reactive oxygen species” OR ROS OR “oxidative stress”); (Q2) (“mitochondrial ROS” OR mtROS OR “mitochondrial reactive oxygen species” OR “mitochondrial DNA”) AND NLRP3 AND (obesity OR “adipose tissue” OR “insulin resistance”); (Q3) TXNIP AND NLRP3 AND (obesity OR “insulin resistance” OR diabetes OR “adipose tissue”); (Q4) NLRP3 AND (“adipose tissue” OR adipocyte*) AND (“insulin resistance” OR obesity) AND (human OR patients OR subjects); (Q5) (“NLRP3 inhibitor” OR “NLRP3 inhibitors” OR MCC950 OR dapansutrile OR OLT1177 OR selnoflast OR DFV890 OR ZYIL1 OR NT-0796 OR ruvonoflast) AND (obesity OR “insulin resistance” OR metabolic OR diabetes); and (Q6) (metformin OR “SGLT2 inhibitor” OR “SGLT2 inhibitors” OR empagliflozin OR “GLP-1 receptor agonist” OR “GLP-1 receptor agonists” OR liraglutide OR semaglutide) AND NLRP3 AND (“oxidative stress” OR ROS OR “reactive oxygen species”). These combinations identified 181, 47, 175, 77, 216, and 134 records, respectively (830 in total). After the removal of 81 duplicates across combinations, 749 unique records remained. Record counts were reported for PubMed only because the Scopus and Web of Science searches were not rerun for this revision. Priority was given to (1) mechanistic studies directly testing ROS/mtROS–NLRP3 links; (2) human adipose tissue, observational, and interventional studies; (3) genetic or pharmacological loss-of-function studies, including those with negative or contradictory findings; (4) clinical studies of NLRP3 inhibitors, IL-1 blockade, and established metabolic drugs; and (5) recent (2023–2026) primary reports and reviews. Additional records were identified from the reference lists of key articles and reviews, targeted searches for foundational mechanistic studies and clinical trials, and ClinicalTrials.gov (accessed 24 September 2026). Because study selection followed a narrative rather than a PRISMA-based systematic process, the number of records screened and excluded at each stage has not been reported; instead, the contribution of the reproducible search to the final reference list is given. Of the 154 references cited, 17 were among the 749 unique PubMed records (matched by DOI or title); the remaining references were incorporated through original literature searches, reference-list screening, and targeted searches for foundational mechanistic studies and clinical trials. Both in vitro and in vivo experimental studies and human studies published in English were considered; conference abstracts, non-peer-reviewed articles, and studies unrelated to obesity or metabolic inflammation were excluded. No formal risk-of-bias assessment or quantitative synthesis was performed. New unpublished data are reported. Figure 1, Figure 2, Figure 3, Figure 4 and Figure 5 were redrawn for this revision using Python (version 3.14.3) with the Matplotlib library (version 3.11.0).

## 3. Oxidative Stress and Adipose Tissue: Setting the Stage

### 3.1. Redox Imbalance and Sources of ROS in Obesity

Oxidative stress arises when the generation of reactive species exceeds the antioxidant buffering capacity, resulting in cellular and tissue damage [12,13]. Oxidants are classified by their primary reactive atom into reactive oxygen species (ROS), reactive nitrogen species (RNS), reactive sulfur species, and reactive chlorine species, of which ROS are the most abundant [14]. ROS include superoxide anion (O_2_•^−^), hydrogen peroxide (H_2_O_2_), and hydroxyl radical (•OH), formed largely through partial reduction of oxygen during cellular metabolism [15,16]. In obesity, excess chronic nutrients amplify ROS production from several endogenous sources, including mitochondrial electron transport, NADPH oxidases (NOX1–5), endoplasmic reticulum (ER) stress, and peroxisomal fatty acid oxidation, while environmental exposures add an exogenous load [8]. Sustained overproduction overwhelms antioxidant defenses (superoxide dismutase, catalase, and glutathione peroxidase) and establishes a self-reinforcing cycle of redox imbalance and inflammation, which impairs metabolic regulation [17,18]. Recent work indicates that adiposity itself enhances systemic oxidative stress and that this burden tracks with cardiometabolic risk, thereby supporting ROS as an early and potentially modifiable contributor rather than only a terminal consequence [6,7].

Among these, mitochondria are a major source of pathological ROS in metabolic tissue. Electron leaks in complexes I and III generate superoxide, which is normally controlled by SOD1/SOD2. Under nutrient overload, this control is exceeded, and mtROS accumulate [19,20]. As described in Section 4, mtROS are not merely a marker of mitochondrial dysfunction, but one of the most consistently implicated signals for NLRP3 activation, placing mitochondria at a central position in the ROS–NLRP3 axis [21,22].

### 3.2. Adipose Tissue as an Oxidative and Inflammatory Organ

Adipose tissue is a heterogeneous endocrine organ comprising adipocytes, preadipocytes, macrophages, endothelial cells, and lymphocytes, and is a major site of oxidative and nitrosative stress in obesity (Figure 1) [23,24,25]. Beyond energy storage, adipocytes secrete adipokines that regulate insulin sensitivity, lipid metabolism, and inflammation. In obesity, adipokine dysregulation and expansion-associated hypoxia promote a pro-inflammatory, pro-oxidant milieu [26,27]. Under chronic energy surplus, adipocyte hypertrophy, infiltration by pro-inflammatory macrophages, and excess glucose/lipid flux increase ROS and RNS generation, which in turn activate stress-responsive pathways including NF-κB, p38 MAPK, JNK, and the hexosamine pathway [28,29,30,31].

The multifactorial etiology of obesity: genetic (monogenic leptin–melanocortin defects and polygenic risk), dietary and energy-balance factors, family history and lifestyle, and gut microbiome dysbiosis, has been well established and reviewed extensively [32,33,34,35,36,37,38,39,40,41,42,43,44,45,46,47,48,49,50,51,52,53,54,55,56,57]. These factors converge on a common downstream consequence relevant to this review: the expansion of dysfunctional, hypoxic adipose tissue that amplifies ROS production and primes inflammatory signaling. Therefore, we treated the obesity etiology as an upstream context and, for the remaining analysis, focused on how the resulting oxidative load was transduced into NLRP3-driven inflammation and insulin resistance.

### 3.3. From Oxidative Damage to Signaling: A Causal Question

ROS exert dual effects: at physiological levels, they serve as signaling intermediates, whereas sustained excess damages lipids, proteins, and DNA and drives disease [58,59]. Membrane polyunsaturated fatty acids are particularly vulnerable, and lipid peroxidation generates reactive carbonyl species that propagate injury and can trigger regulated cell death [60,61,62]. A central and still unresolved question is whether ROS are the primary causal mediators of adipocyte insulin resistance or secondary amplifiers of an already perturbed metabolic state [63]. Landmark work in cultured adipocytes and mice demonstrated that ROS play a causal role in multiple forms of insulin resistance, supporting the former [8]. However, the inconsistent metabolic effects of antioxidant supplementation in humans caution against a simple linear model. This tension, causal driver versus amplifier, recurs throughout the ROS–NLRP3 literature and is critically examined in Section 5.

## 4. ROS–NLRP3 Axis in Obesity

Having established ROS as a convergent oxidative signal of dysfunctional adipose tissue, this section develops the central argument of the review: ROS contribute to two separate steps of NLRP3 regulation: priming (signal 1), in which ROS-responsive NF-κB signaling increases the expression of inflammasome components (Section 4.1), and activation (signal 2), in which mtROS and related mitochondrial signals are implicated in inflammasome assembly (Section 4.2 and Section 4.3). The p38 MAPK and hexosamine pathways are discussed as parallel ROS- or nutrient-responsive pathways that may reinforce the inflammatory insulin-resistant state rather than as components of NLRP3 priming. The integrated axis, from ROS sources through NLRP3 to insulin resistance and obesity progression, was developed in the following subsections and consolidated in an integrated summary figure presented with the conclusions.

### 4.1. ROS-Responsive Signaling Pathways Priming Inflammation

**NF-κB signaling:** NF-κB is the principal transcriptional gateway for inflammatory gene expression and, critically, is a major route of the priming signal (signal 1) for NLRP3 [64]. In obesity-associated oxidative stress, ROS together with TNF and IL-1 can activate the canonical pathway: receptor engagement activates the IκB kinase (IKK) complex, which phosphorylates IκBα, triggering its ubiquitination and proteasomal degradation and releasing NF-κB dimers to induce the transcription of pro-inflammatory cytokines and of NLRP3, pro-IL-1β, and pro-IL-18 [65,66]. Sustained IKK–NF-κB activity also promotes inhibitory serine phosphorylation of insulin receptor substrate-1 (IRS-1), thus linking inflammatory signaling to insulin resistance (Figure 2) [66,67].

**p38 MAPK signaling:** ROS are important upstream activators of MAPK cascades. A well-characterized redox mechanism operates through apoptosis signal-regulating kinase 1 (ASK1): under basal conditions, ASK1 is held inactive by reduced thioredoxin, but oxidation of thioredoxin during oxidative stress releases and activates ASK1, driving the JNK and p38 MAPK pathways that contribute to inflammation, apoptosis, and metabolic dysfunction (Figure 3) [68,69,70,71,72]. Notably, this thioredoxin-oxidation switch parallels the proposed TXNIP-based activation of NLRP3, as discussed below, suggesting that thioredoxin redox status is a possible shared upstream sensor.

**Hexosamine biosynthetic pathway (HBP):** HBP is a nutrient-sensing branch of glucose metabolism whose flux increases in overnutrition and diabetes [73]. Its product, UDP-GlcNAc, drives O-GlcNAcylation of insulin signaling components, impairing GLUT4 translocation and contributing to insulin resistance [74,75,76,77]. Because HBP flux integrates glucose, lipid, and amino acid availability, it links nutrient overload to the same insulin-resistant, pro-inflammatory state reinforced by ROS [78].

### 4.2. NLRP3 Inflammasome: Structure and the Two-Signal Model

The NLRP3 inflammasome is a cytosolic multiprotein complex comprising the sensor NLRP3, the adaptor ASC, and pro-caspase-1 [9]. Of the 22 NLR members identified (NLRP1–NLRP14 and related receptors), NLRP3 is the most strongly tied to obesity, insulin resistance, and mtROS generation [79,80]. Upon activation, NLRP3 nucleates ASC, whose caspase recruitment domain (CARD) engages pro-caspase-1; autocatalytic cleavage yields active caspase-1, which matures IL-1β and IL-18 and can execute gasdermin-D-dependent pyroptosis [79,81,82,83]. The inflammasome responds to danger- and pathogen-associated molecular patterns (DAMPs/PAMPs) sensed by pattern recognition receptors, and metabolic DAMPs are central to its role in obesity [84,85].

Activation follows a two-signal model (Figure 4) [10,86]. **Signal 1 (priming)** is provided by NF-κB-activating stimuli (TLR ligands, TNF, endogenous mediators) that transcriptionally upregulate NLRP3, pro-IL-1β, and pro-IL-18; MyD88–IRAK1/IRAK4 mediate transcriptional priming while non-transcriptional licensing involves deubiquitination of NLRP3 by BRCC36 [86,87,88,89,90,91]. Priming increases the availability of inflammasome components but does not by itself trigger inflammasome assembly. **Signal 2 (activation)** is triggered by potassium efflux, calcium flux, lysosomal destabilization with cathepsin B release, mitochondrial dysfunction, and ROS [92,93,94,95,96,97,98,99,100,101]. Whether K^+^ efflux is the dominant obligatory event or whether Ca^2+^ signaling contributes independently remains debated, reflecting genuine mechanistic uncertainty rather than settled consensus [96,97].

### 4.3. mtROS as a Major Proximal Signal for NLRP3 Activation

Mitochondrial ROS are among the most consistently implicated signals for NLRP3 activation, although the requirement for mitochondria has been questioned and appears to depend on the stimulus [102,103]. Three mechanistically distinct routes have been proposed to link ROS and mitochondrial signals to NLRP3, which are largely defined in macrophages rather than in adipocytes or other metabolic cells. First, early studies with particulate activators showed that ROS, generated by NADPH oxidase in that setting, were required for NLRP3 (Nalp3) activation by asbestos and silica [104]. Second, oxidized mitochondrial DNA released into the cytosol during apoptosis binds to NLRP3 and promotes its activation, and the loss of autophagy proteins enhances mitochondrial DNA release and NLRP3-dependent caspase-1 activation in macrophages [105,106]. Third, thioredoxin-interacting protein (TXNIP) has been proposed to dissociate from oxidized thioredoxin under high ROS/high glucose conditions and bind NLRP3, providing a redox- and glucose-sensitive activation switch of potential relevance to hyperglycemic and obese states [107]. Additional mitochondrial contributors—cardiolipin externalization to the outer membrane and MAVS-dependent recruitment—have been proposed but remain of uncertain physiological weight and are regarded as unresolved [108,109,110].

The therapeutic corollary is that interventions that lower mtROS levels may attenuate NLRP3 activation upstream of the inflammasome itself, a possibility that remains to be tested in humans. A review of metabolism–NLRP3 interactions has highlighted mtROS and metabolic licensing as potential upstream targets [111], and in a high-fat diet mouse model of obesity-associated cardiomyopathy, the mitochondria-targeted antioxidant MitoTEMPO was used to probe mtROS, with increased mtROS and TXNIP linked to excessive NLRP3 inflammasome activation [112]. This positions mtROS as a potentially relevant proximal node of the axis, although direct evidence for human intervention is lacking.

### 4.4. Downstream Consequences: From IL-1β/IL-18 to Insulin Resistance

Mature IL-1β and IL-18 released from adipose macrophages and adipocytes sustain a paracrine inflammatory loop: they recruit and polarize macrophages, suppress adiponectin, and, through NF-κB and JNK, promote IRS-1 serine phosphorylation that blunts PI3K/AKT signaling and GLUT4 translocation, impairing glucose uptake [66,113,114]. Human data are consistent with clinical relevance but remain mainly observational. In a case–control study of 98 subjects, obesity, obesity-associated T2DM, and NAFLD (now termed MASLD) were associated with increased expression of inflammasome components in adipose tissue, and obese patients with T2DM showed higher hepatic NLRP3, IL1B, and IL18 expression [115]. In visceral adipose tissue from 23 metabolically unhealthy obese, 21 metabolically healthy obese, and nine lean individuals, the metabolically unhealthy phenotype was associated with higher IL-1β secretion, higher NLRP3 and IL1B expression, and greater caspase-1 activation in adipose tissue macrophages [116]. Subcutaneous adipose CASPASE-1 expression was higher in 10 obese men with impaired glucose tolerance than in nine lean age-matched controls and was associated with insulin resistance [117], and in 58 obese adolescents, a high visceral-to-total abdominal fat ratio was associated with increased expression of inflammasome-related genes in subcutaneous adipose tissue [118]. In monocyte-derived macrophages, NLRP3 inflammasome activation and mtROS-dependent IL-1β processing were increased in 47 drug-naïve patients with newly diagnosed T2DM compared with 57 controls [119]. These studies show associations and do not establish causality. The resulting insulin resistance and adipose dysfunction may further increase metabolic stress and mtROS levels, forming a proposed feed-forward loop (Figure 5) that could entrench obesity progression, which is supported mainly by preclinical data. This self-amplifying character is the conceptual core of the review: ROS and NLRP3 are not sequential endpoints, but a coupled circuit.

## 5. Critical Appraisal: Evidence, Controversies, and Knowledge Gaps

A rigorous evaluation of the ROS–NLRP3 axis must account for the inconsistent findings reported in the literature. Several foundational studies have established that inflammasome deficiency protects against diet-induced obesity and insulin resistance; mice lacking Nlrp3, ASC, or caspase-1 resist high-fat diet-induced obesity and its metabolic sequelae, and NLRP3 senses obesity-associated danger signals to drive IL-18 and adipose T-cell inflammation [120,121]. In the latter study, subcutaneous adipose NLRP3 and IL1B expression also decreased in 10 obese men with T2DM after a 1-year lifestyle weight-loss intervention, although this before–after comparison had no control group [121]. However, an equally credible body of loss-of-function studies reported the opposite. Nlrp3-null mice were not protected from Western-diet-induced adipose inflammation or glucose intolerance in one study (*n* = 8 per group) [122], and genetic deletion or pharmacological inhibition of Nlrp3 failed to ameliorate experimental NASH (now termed metabolic dysfunction-associated steatohepatitis, MASH) induced by a high-fat, high-cholesterol diet [123]. These discordant results are not trivial; they indicate that the metabolic contribution of NLRP3 is conditional on genetic background, diet composition, tissue, disease stage, and microbiome, rather than being a fixed, linear driver.

The therapeutic literature adds further nuances. The prototypical selective NLRP3 inhibitor MCC950 (CRID3) reduces adipose and cardiac inflammation in obese models, but in an interventional model of diabetic kidney disease in streptozotocin-diabetic ApoE^−^/^−^ mice it was associated with adverse renal effects [124], and reported hepatotoxicity halted its early clinical development [125]—cautioning against the assumption that inflammasome suppression is uniformly beneficial. This has motivated next-generation and brain-penetrant inhibitors and clinical candidates developed through phenotypic screening, which were designed to improve selectivity and safety [125,126].

Table 1 contrasts representative supporting and contradictory studies across model, intervention, and outcome, making explicit where the evidence converges and where it conflicts.

Three knowledge gaps emerge from this appraisal. **First, causality and context:** much mechanistic evidence derives from rodent loss-of-function models whose discordant findings suggest the axis is modifier-dependent; human causal data remain limited, consisting mainly of cross-sectional tissue studies and small uncontrolled interventions [115,116,117,118,119,121,127,128]. **Second, tissue and stage specificity:** NLRP3’s role appears to differ between adipose tissue, liver, kidney, and vasculature, and between early and advanced disease; however, most reviews treat it monolithically. **Third, the dual physiology of ROS and NLRP3:** because both are essential for normal host defense and signaling, sustained global suppression risks off-target harm, as the MCC950 safety signals illustrate [124]. Reconciling these gaps requires human tissue studies, biomarker-stratified interventions, and tissue-selective pharmacology rather than further confirmatory rodent reports.

## 6. Therapeutic Implications and Future Directions

The context-dependent nature of the ROS–NLRP3 axis suggests that therapy is best conceptualized at three levels: upstream (ROS generation), core (inflammasome assembly), and downstream (cytokine effectors), rather than as a single target. Figure 5 maps candidate interventions onto these levels, and Table 2 indicates the stage of evidence supporting each strategy (in vitro, animal, human tissue/observational, early clinical, or clinical outcome). Established metabolic drugs are considered separately because ROS/NLRP3 modulation is, at most, a contributory mechanism of their clinical effects.

**Table 2:** Therapeutic strategies mapped onto the three levels of the ROS–NLRP3 axis in obesity—upstream (ROS generation), core (inflammasome assembly), and downstream (cytokine effectors)—with established metabolic drugs listed separately as contributory modulators.

Axis Level (Node)	Strategy/Agent	Effect on the ROS–NLRP3 Axis	Evidence Stage	Reference
Upstream (mtROS)	Mitochondria-targeted antioxidants (MitoTEMPO/Mito-Tempo)	Mito-Tempo ↓ NLRP3 activation and pyroptosis via Nrf2 reactivation (ox-LDL macrophages); mtROS and TXNIP linked to NLRP3 activation (obese cardiomyopathy mice; MitoTEMPO used as probe)	Animal (HFD obese cardiomyopathy mice) + in vitro (NRVMs; ox-LDL macrophages)	[112,129]
Upstream (TXNIP)	TXNIP blockade/redox-glucose switch	NAC abolishes ROS-dependent TXNIP–NLRP3 proximity; NAC or MCC950 restores insulin-dependent glucose uptake in muscle fibers	Review + animal (HFD insulin-resistant mice)	[107,130]
Upstream (NOX/global redox)	Nutraceutical antioxidants (melatonin, resveratrol, vitamins C/D/E, polyphenols, flavonoids, curcumin, n-3 PUFAs, EGCG, others)	Scavenge ROS, ↑ SOD/GPx/catalase, inhibit NOX, ↓ NF-κB/MAPK priming; inconsistent clinical efficacy (bioavailability, non-targeted)	Reviews (preclinical + mixed clinical data)	[7,16,23,25]
Upstream (lifestyle)	Caloric restriction/weight loss	↓ PBMC mtROS, ↑ glutathione, ↑ mitochondrial respiration; ↓ SAT NLRP3/IL1B expression after weight loss	Human (uncontrolled before–after; NLRP3 not assessed in [128])	[121,128]
Core (NLRP3 assembly)	Selective NLRP3 inhibitor—MCC950 (benchmark)	Blocks NLRP3 activation; ↓ adipose/cardiac inflammation; organ-specific toxicity limits use	Animal (clinical development halted)	[124,125]
Core (NLRP3 assembly)	Next-generation/clinical-stage NLRP3 inhibitors (dapansutrile, selnoflast, NT-0796/ruvonoflast, DFV890, ZYIL1)	Selective NLRP3 inhibition, designed to improve selectivity and safety; ↓ hsCRP with ruvonoflast in an obesity-enriched phase 1b trial; no clinical outcome data in obesity	Early clinical (phase 1–2a; mostly non-obesity indications)	[125,126,131,132,133,134,135,136,137]
Downstream (IL-1β/IL-18)	Cytokine blockade (anti-IL-1β/IL-18)	Neutralizes mature effectors; anakinra ↓ HbA1c in T2DM (RCT); canakinumab ↓ recurrent CV events (CANTOS); not obesity-specific	Early clinical (RCT, T2DM) + clinical outcome (CANTOS)	[10,66,138,139]
Contributory (established metabolic drug)	Metformin	Clinical benefit: UKPDS 34 (ROS–NLRP3 not assessed). Mechanism: ↓ VAT NLRP3, ASC, NF-κB and leukocyte mtROS (cross-sectional); ↓ macrophage IL-1β maturation via AMPK (uncontrolled, *n* = 11)	Clinical outcome (benefit); human observational + small uncontrolled study + review (mechanism)	[119,127,140,141]
Contributory (established metabolic drug)	SGLT2 inhibitors (empagliflozin, dapagliflozin, canagliflozin)	Clinical benefit: EMPA-REG OUTCOME (ROS–NLRP3 not assessed). Mechanism: ↓ macrophage IL-1β secretion vs. glimepiride (open-label RCT, 30 days); thioredoxin–NLRP3-dependent ↓ pyroptosis (rat)	Clinical outcome (benefit); early clinical + animal + review (mechanism)	[142,143,144,145,146]
Contributory (established metabolic drug)	GLP-1 receptor agonists (liraglutide, semaglutide)	Clinical benefit: SELECT (ROS–NLRP3 not assessed). Mechanism: ↓ leukocyte ROS, IL-6, TNF-α (cross-sectional; NLRP3 not measured); ↓ NLRP3/ASC/caspase-1 and pyroptosis or oxidative stress (podocytes, endothelial cells; in vitro)	Clinical outcome (benefit); human observational + in vitro (mechanism)	[147,148,149,150]

For each strategy, the axis node engaged and the reported effects on ROS/NLRP3 signaling are indicated, with representative agents and evidence. The evidence stage is classified as in vitro, animal, human tissue/observational, early clinical, or clinical outcomes. For established metabolic drugs, clinical benefit evidence and mechanistic evidence are listed separately. Arrows indicate the direction of change: ↑, increase/upregulation; ↓, decrease/downregulation. Abbreviations: mtROS, mitochondrial reactive oxygen species; TXNIP, thioredoxin-interacting protein; NOX, NADPH oxidase; SOD, superoxide dismutase; GPx, glutathione peroxidase; Nrf2, nuclear factor erythroid 2-related factor 2; NF-κB, nuclear factor-κB; MAPK, mitogen-activated protein kinase; ASC, apoptosis-associated speck-like protein; IL, interleukin; TNF-α, tumor necrosis factor-α; AMPK, AMP-activated protein kinase; SGLT2, sodium–glucose cotransporter 2; GLP-1, glucagon-like peptide-1; T2DM, type 2 diabetes mellitus; VAT, visceral adipose tissue; SAT, subcutaneous adipose tissue; NAC, N-acetylcysteine; PBMC, peripheral blood mononuclear cell; hsCRP, high-sensitivity C-reactive protein; RCT, randomized controlled trial; CV, cardiovascular.

### 6.1. Upstream: Attenuating ROS Generation

Because mtROS are implicated to be proximal to NLRP3 activation, reducing the mitochondrial oxidative burden is mechanistically attractive. In high-fat-diet mice with obesity-associated cardiomyopathy and in palmitate-treated neonatal rat ventricular myocytes, increased mtROS and TXNIP were linked to excessive NLRP3 activation, and the mitochondria-targeted antioxidant MitoTEMPO was used to probe this pathway [112]; in ox-LDL-treated macrophages in vitro, the related compound Mito-Tempo suppressed NLRP3 inflammasome activation and pyroptosis by reactivating Nrf2 [129]. These data support mitochondrial ROS as a potentially druggable upstream node, although only in animal and in vitro models. TXNIP is an appealing target because it couples glucose and redox status to inflammasome activation [107]. A 2024 study showed that oxidative stress increases TXNIP–NLRP3 proximity in skeletal muscle fibers of mice made insulin resistant by 8 weeks of high-fat feeding, and that N-acetylcysteine or MCC950 restored insulin-dependent glucose uptake [130]. Classical and nutraceutical antioxidants—vitamins C, D, and E, polyphenols, flavonoids, n-3 PUFAs, and plant-derived extracts—scavenge ROS, upregulate SOD/catalase/glutathione peroxidase, or inhibit NOX enzymes, and are summarized in Table 2. However, their clinical efficacy in obesity has been inconsistent, plausibly reflecting poor bioavailability, lack of mitochondrial targeting, and the dual signaling role of ROS; non-targeted global antioxidants may blunt beneficial redox signaling along with pathological ROS. This suggests compartment-specific rather than systemic antioxidant strategies. In humans, an uncontrolled 24-week program of very-low-calorie and low-calorie diets in 109 subjects with obesity reduced mtROS and increased glutathione in peripheral blood mononuclear cells while improving mitochondrial respiration, but the circulating interleukin profile, including IL-1β, did not improve, and NLRP3 was not assessed [128]. Together with the weight loss data described in Section 5 [121], these findings suggest lifestyle intervention as a plausible physiological upstream modulator of the axis. However, controlled studies with NLRP3 readouts are lacking.

### 6.2. Core: Selective NLRP3 Inhibition

Direct NLRP3 inhibitors offer target specificity. Although MCC950 remains the pharmacological benchmark in preclinical studies, its reported hepatotoxicity and adverse renal effects in a diabetic kidney disease model highlight the need for safer compounds with broader therapeutic windows [124,125]. Several next-generation inhibitors designed to improve selectivity and safety have entered early clinical development. However, human evidence is limited to phase 1–2a studies, mostly outside obesity. Dapansutrile (OLT1177) was safe in healthy volunteers receiving 1000 mg daily for 8 days [131], and in an open-label, dose-adaptive phase 2a trial in patients with gout flares (34 enrolled; 29 per protocol), reduced joint pain with satisfactory safety [132]. Selnoflast was safe in a randomized, double-blind, placebo-controlled phase 1b study in 19 patients with ulcerative colitis; however, pharmacodynamic biomarkers showed no robust differences compared to placebo [133]. DFV890 was tested in an early phase 2a randomized trial of 143 patients with COVID-19 pneumonia, in which the primary endpoint was not met [134]. ZYIL1 expression was evaluated in the first-in-human single-ascending-dose (*n* = 30) and multiple-ascending-dose (*n* = 18) studies in healthy subjects [135]. Ruvonoflast (NT-0796) is, to our knowledge, the only inhibitor with published data in an obesity-enriched population: in a double-blind phase 1b trial in adults with a body-mass index of 30–40 kg/m^2^, high-sensitivity C-reactive protein (hsCRP) ≥ 2.5 mg/L, and cardiometabolic comorbidity, 28 days of ruvonoflast (225 mg twice daily; *n* = 40) reduced hsCRP by 82.2% versus 37.2% with placebo (*n* = 23), with comparable changes in body weight [136]. A phase 2 placebo-controlled trial of dapansutrile in T2DM (ClinicalTrials.gov NCT06047262) was listed as recruiting when accessed on 24 September 2026, and none of these agents have yet been evaluated in clinical outcome trials in obesity. Brain-penetrant compounds are also being developed for neuroinflammatory indications [125,126,137]. Their success depends on tissue selectivity and on identifying patients whose disease is genuinely NLRP3-dependent, precisely the stratification that current correlative human data cannot yet provide.

### 6.3. Downstream: Cytokine Blockade and Established Metabolic Drugs as Contributory Modulators

Blockade of IL-1 signaling has clinical evidence, although not specifically in obesity. In a randomized, placebo-controlled trial in 70 patients with T2DM, 13 weeks of the IL-1 receptor antagonist anakinra lowered HbA1c by 0.46 percentage points more than placebo, without changes in insulin resistance or body-mass index [138], and in the CANTOS trial of 10,061 patients with previous myocardial infarction and hsCRP ≥ 2 mg/L, the anti-IL-1β antibody canakinumab (150 mg) reduced recurrent cardiovascular events (hazard ratio 0.85) independently of lipid lowering [139]. Several established metabolic drugs also have proven clinical benefit, metformin in overweight patients with T2DM in UKPDS 34 [140], empagliflozin in patients with T2DM at high cardiovascular risk in EMPA-REG OUTCOME [142], and semaglutide in 17,604 adults with overweight or obesity and cardiovascular disease but without diabetes in SELECT [147]; however, ROS–NLRP3 signaling was not assessed in these outcome trials, and their benefits cannot be attributed to this axis. Mechanistic evidence for these drugs is therefore considered separately. For metformin, a cross-sectional study of patients undergoing bariatric surgery revealed lower visceral adipose NLRP3, ASC, and NF-κB, and lower leukocyte mtROS in metformin-treated obese patients with T2DM (*n* = 34) than in metabolically healthy obese individuals (*n* = 34). However, metformin dose and duration were not reported [127]; in an uncontrolled study of 11 patients with T2DM, 8 weeks of metformin reduced IL-1β maturation in monocyte-derived macrophages through AMPK activation [119]; and a recent review links these effects to NF-κB/NEK7/AMPK signaling and mitochondrial function [141]. For SGLT2 inhibitors and GLP-1 receptor agonists, anti-inflammatory and antioxidant effects that extend beyond glycemic control have been reported [143,148]. In a randomized, open-label, active-controlled trial in patients with T2DM at high cardiovascular risk (71 randomized; 61 completed), 30 days of empagliflozin reduced macrophage IL-1β secretion more than glimepiride, in association with increased serum β-hydroxybutyrate and decreased serum insulin [144]. Other SGLT2 inhibitor data were obtained from a review of the inflammasome pathway in acute myocardial infarction [145] and from a rat high-fat cholesterol diet/streptozotocin model, in which empagliflozin attenuated pyroptosis through the thioredoxin–NLRP3 axis [146]. For GLP-1 receptor agonists, a cross-sectional study found lower leukocyte ROS production and lower IL-6 and TNFα levels in treated (*n* = 59) than in untreated (*n* = 196) patients with T2DM but did not measure NLRP3 [148]; NLRP3/ASC/caspase-1 suppression and reduced pyroptosis or oxidative stress have been shown in mouse podocytes and human endothelial cells in vitro [149,150]. Taken together, ROS–NLRP3 modulation is a potentially relevant, hypothesis-generating contributory mechanism of these drugs rather than an established basis for their clinical benefits and provides a rationale for mechanistic readouts in future clinical studies.

### 6.4. Future Directions

Future research priorities emerge directly from the knowledge gaps discussed in Section 5: (i) **Human causal validation:** intervention trials with mechanistic readouts (adipose NLRP3 activity, mtROS, and IL-1β/IL-18) rather than correlative associations. (ii) **Patient stratification:** biomarkers that identify NLRP3-dependent disease subsets, given the modifier-dependent rodent discordance. (iii) **Tissue-selective delivery:** mitochondria-targeted and adipose- or liver-directed formulations to exploit the axis while preserving protective ROS/NLRP3 function. (iv) **Combination strategies:** pairing upstream mtROS control with downstream cytokine or metabolic drug effects and integrating lifestyle interventions. (v) **Resolving mechanistic uncertainties:** the physiological weight of cardiolipin, MAVS, and Ca^2+^ versus K^+^ efflux in metabolic tissue. Addressing these issues will help determine whether the ROS–NLRP3 axis can move from a compelling mechanistic framework to validated clinical practice.

## 7. Conclusions

Obesity is a state of chronic low-grade inflammation in which ROS act not only as damaging byproducts but also as signaling contributors. This review has argued that ROS contribute to NLRP3 regulation at two separable steps: ROS-responsive NF-κB signaling can support priming, whereas mitochondrial signals mtROS, oxidized mtDNA, and TXNIP are consistently implicated in activation, leading to IL-1β/IL-18-mediated inflammation and insulin resistance that may, in turn, sustain metabolic stress (Figure 5). Although preclinical evidence for this model is substantial, human causal evidence remains limited. Available human data consist mainly of cross-sectional tissue studies and small uncontrolled interventions, and credible loss-of-function studies report no metabolic protection. Therefore, the ROS–NLRP3 axis is best regarded as a conditional, tissue-, stage-, and context-dependent circuit rather than a universal linear pathway, and its contribution to obesity progression requires validation in human mechanistic and interventional studies.

Therapeutic implications should be framed with the same caution. Upstream mtROS control (including mitochondria-targeted antioxidants and lifestyle) and core selective NLRP3 inhibition are supported mainly by preclinical and early clinical data; downstream IL-1 blockade has clinical evidence, although not specifically in obesity; for metformin, SGLT2 inhibitors, and GLP-1 receptor agonists, ROS/NLRP3 modulation is a potentially relevant contributory mechanism rather than an established basis of their clinical benefit. Mechanism-based, tissue-selective, and biomarker-stratified human studies are needed to determine whether targeting the axis translates into clinical benefits. Because oxidative stress has also been implicated in COVID-19 [151], neurodegenerative disease [152], and cancer [153], mitochondrial stress–NLRP3 coupling has been investigated in lung and kidney diseases [124,154], and both ROS and NLRP3 contribute to host defense, non-selective suppression carries a risk of off-target harm.

## Figures and Tables

**Table 1:** Critical comparison of key studies on the NLRP3 inflammasome in obesity and metabolic disease, including supporting and contradictory findings. Sample sizes are given as reported in the original articles; NR, not reported.

Study/Model	Species	Sample Size (*n*)	Study Design	Intervention/Exposure	Key Metabolic Outcome	Direction of Evidence	Reference
Stienstra et al., 2011; inflammasome-deficient mice	Mouse (*Nlrp3*^−^/^−^, *Asc*^−^/^−^, *Casp1*^−^/^−^; C57BL/6 background)	6–8 per group	Genetic LOF	HFD	Resistance to diet-induced obesity and insulin resistance; ↓ hepatic TG, adipocyte size, and AT macrophage infiltration	Supporting (animal)	[120]
Vandanmagsar et al., 2011; diet-induced obesity	Mouse (*Nlrp3*^−^/^−^ vs. WT)	4–10 per group (by endpoint)	Genetic LOF	60% HFD	↓ IL-18, ↓ adipose IFNγ; improved insulin signaling	Supporting (animal)	[121]
Vandanmagsar et al., 2011; human weight-loss substudy	Human (obese men with T2DM; SAT biopsies)	10	Uncontrolled before–after	1-year lifestyle weight-loss intervention	↓ SAT NLRP3 and IL1B expression, with lower glycemia and improved HOMA-IR	Supporting (human; association)	[121]
Unamuno et al., 2021; human VAT/liver and visceral adipocytes	Human	98 (VAT gene expression: 10 lean, 30 obese NG, 30 obese IGT/T2DM); in vitro, 6 per group	Case–control + in vitro siRNA	Obesity, T2DM, NAFLD; NLRP3 knockdown	↑ inflammasome components and IL-1β/IL-18 in AT with obesity, T2DM, NAFLD; ↑ hepatic NLRP3, IL1B, IL18 in obese T2DM; knockdown ↓ LPS-induced adipocyte inflammation and fibrosis genes	Supporting (human; association + in vitro)	[115]
Esser et al., 2013; SAT and VAT	Human	23 MUO, 21 MHO, 9 lean	Cross-sectional	Metabolically unhealthy vs. healthy obesity	↑ VAT IL-1β secretion, NLRP3 and IL1B expression, and ATM caspase-1 activation in MUO	Supporting (human; association)	[116]
Goossens et al., 2012; SAT	Human	10 obese men with IGT, 9 lean age-matched controls	Cross-sectional	Obesity with IGT	↑ SAT CASPASE-1 expression, associated with insulin resistance	Supporting (human; association)	[117]
Kursawe et al., 2016; SAT of obese adolescents	Human (adolescents)	58	Cross-sectional	High vs. low visceral-to-total abdominal fat ratio	↑ SAT inflammasome-related gene expression with high visceral fat partitioning	Supporting (human; association)	[118]
Lee et al., 2013; MDMs	Human	47 T2DM vs. 57 controls; 11 treated with metformin	Cross-sectional + uncontrolled before–after	Newly diagnosed T2DM; metformin 500–1000 mg/day, 8 weeks	↑ NLRP3 activation and mtROS-dependent IL-1β processing in T2DM; ↓ IL-1β maturation after metformin (AMPK)	Supporting (human; association)	[119]
Liu et al., 2024; obesity-associated cardiomyopathy	Mouse (male WT, NLRP3^−^/^−^); NRVMs	5–12 per group (by endpoint)	Genetic LOF + pharmacological	HFD, 24 weeks; MCC950; MitoTEMPO; TXNIP overexpression; palmitate	NLRP3 deletion or MCC950 prevented cardiac dysfunction, hypertrophy, fibrosis, and lipid accumulation; mtROS and TXNIP linked to NLRP3 activation	Supporting (animal; upstream mtROS)	[112]
Ringling et al., 2016; Western diet	Mouse (male C57BL/6J WT vs. *Nlrp3*^−^/^−^)	8 per group	Genetic LOF	Western diet (45% fat, 1% cholesterol), 24 weeks	No protection from adipose inflammation or glucose intolerance; no ↑ caspase-1 cleavage	Contradictory (animal)	[122]
Ioannou et al., 2023; experimental NASH	Mouse (male *Nlrp3* KO, *Casp1* KO, WT)	12 per group (genetic arm); 24 and 18 mice (pharmacological arm)	Genetic LOF + pharmacological	High-fat, high-cholesterol diet, 6 months; MCC950 in drinking water	Did not ameliorate NASH	Contradictory (animal)	[123]
Østergaard et al., 2022; diabetic kidney disease	Mouse (male STZ-diabetic ApoE^−^/^−^); human podocytes	7–11 per group	Pharmacological intervention	MCC950	Adverse renal effects	Contradictory (safety; animal)	[124]
Abad-Jiménez et al., 2020; VAT and leukocytes of RYGB patients	Human	68 (34 MHO, 34 T2DM on metformin)	Cross-sectional	Metformin treatment (dose and duration NR)	↓ VAT NLRP3, ASC, NF-κB; ↓ leukocyte mtROS	Supporting (human; association)	[127]
Bosch-Sierra et al., 2024; weight loss	Human (subjects with obesity; PBMCs)	109	Uncontrolled before–after	Very-low-calorie alternating with low-calorie diet, 24 weeks	↓ PBMC mtROS, ↑ glutathione, improved mitochondrial respiration; no improvement in circulating interleukins; NLRP3 not assessed	Supporting (human; upstream, indirect)	[128]

Arrows indicate the direction of change: ↑, increase/upregulation; ↓, decrease/downregulation. Original disease terms (NAFLD and NASH) are retained where they describe the original studies. Sample sizes are those reported in the original articles (ranges give the smallest and largest group sizes reported across endpoints), and study design is provided as stated by the original authors or, where not stated, classified by the review authors from the reported methods. Abbreviations: HFD, high-fat diet; TG, triglycerides; IFNγ, interferon-γ; T2DM, type 2 diabetes mellitus; NAFLD, non-alcoholic fatty liver disease (now MASLD, metabolic dysfunction-associated steatotic liver disease); AT, adipose tissue; VAT, visceral adipose tissue; SAT, subcutaneous adipose tissue; TXNIP, thioredoxin-interacting protein; mtROS, mitochondrial reactive oxygen species; NASH, non-alcoholic steatohepatitis (now MASH, metabolic dysfunction-associated steatohepatitis); LOF, loss-of-function; WT, wild-type; KO, knockout; MUO, metabolically unhealthy obese; MHO, metabolically healthy obese; IGT, impaired glucose tolerance; ATM, adipose tissue macrophage; MDM, monocyte-derived macrophage; NRVM, neonatal rat ventricular myocyte; STZ, streptozotocin; PBMC, peripheral blood mononuclear cell; RYGB, Roux-en-Y gastric bypass; NR, not reported.

## Data Availability

No new data were created or analyzed in this study. Data sharing is not applicable to this study.
